# Cuminaldehyde potentiates the antimicrobial actions of ciprofloxacin against *Staphylococcus aureus* and *Escherichia coli*

**DOI:** 10.1371/journal.pone.0232987

**Published:** 2020-05-14

**Authors:** Valério Monteiro-Neto, Cláudio D. de Souza, Laoane F. Gonzaga, Bruna C. da Silveira, Nágila C. F. Sousa, Jaqueline P. Pontes, Deivid M. Santos, Wanessa C. Martins, Jorge F. V. Pessoa, Alexsander R. Carvalho Júnior, Viviane S. S. Almeida, Natália M. T. de Oliveira, Thayla S. de Araújo, Daniele Maria-Ferreira, Saulo J. F. Mendes, Thiago A. F. Ferro, Elizabeth S. Fernandes

**Affiliations:** 1 Programa de Pós-graduação, Universidade Ceuma, São Luís, MA, Brazil; 2 Universidade Federal do Maranhão, São Luís, MA, Brazil; 3 Instituto de Pesquisa Pelé Pequeno Príncipe, Curitiba, PR, Brazil; 4 Faculdades Pequeno Príncipe, Curitiba, PR, Brazil; 5 Department of Parasitology, Universidade de São Paulo, São Paulo, SP, Brazil; Laurentian, CANADA

## Abstract

*Escherichia coli* and *Staphylococcus aureus* are important agents of urinary tract infections that can often evolve to severe infections. The rise of antibiotic-resistant strains has driven the search for novel therapies to replace the use or act as adjuvants of antibiotics. In this context, plant-derived compounds have been widely investigated. Cuminaldehyde is suggested as the major antimicrobial compound of the cumin seed essential oil. However, this effect is not fully understood. Herein, we investigated the *in silico* and *in vitro* activities of cuminaldehyde, as well as its ability to potentiate ciprofloxacin effects against *S*. *aureus* and *E*. *coli*. *In silico* analyses were performed by using different computational tools. The PASS online and SwissADME programmes were used for the prediction of biological activities and oral bioavailability of cuminaldehyde. For analysis of the possible toxic effects and the theoretical pharmacokinetic parameters of the compound, the Osiris, SwissADME and PROTOX programmes were used. Estimations of cuminaldehyde gastrointestinal absorption, blood brain barrier permeability and skin permeation by using SwissADME; and drug likeness and score by using Osiris, were also evaluated The *in vitro* antimicrobial effects of cuminaldehyde were determined by using microdilution, biofilm formation and time-kill assays. *In silico* analysis indicated that cuminaldehyde may act as an antimicrobial and as a membrane permeability enhancer. It was suggested to be highly absorbable by the gastrointestinal tract and likely to cross the blood brain barrier. Also, irritative and harmful effects were predicted for cuminaldehyde if swallowed at its LD50. Good oral bioavailability and drug score were also found for this compound. Cuminaldehyde presented antimicrobial and anti-biofilm effects against *S*. *aureus* and *E*. *coli*.. When co-incubated with ciprofloxacin, it enhanced the antibiotic antimicrobial and anti-biofilm actions. We suggest that cuminaldehyde may be useful as an adjuvant therapy to ciprofloxacin in *S*. *aureus* and *E*. *coli*-induced infections.

## Introduction

Bacteria such as *Escherichia coli* and *Staphylococcus aureus* are important agents of urinary tract infections [1–4]. Both bacteria are frequently detected in patients with indwelling urinary tract devices [1, 5–7]. These primary infections often evolve to severe infectious diseases such as sepsis, which presents high morbidity and mortality. *E*. *coli* is one of the major pathogens of such infections, causing bacteremia in over 6,000 per 100,000 patients per year [8, 9]. In addition, reports indicate *S*. *aureus* is responsible for 21 to 36 cases of bacteremia per 100,000 habitants per year [10, 11] with mortality rates varying from 30 to 70% [12].

The management of such infections mostly relies on antibiotic therapy, with patient survival and recovery largely depending on treatment timing and efficacy. Of note, ciprofloxacin is the most commonly prescribed fluoroquinolone antibiotic for urinary tract infections [13] to which, both *S*. *aureus* and *E*. *coli* have become resistant [3, 14]. Indeed, the unrestricted and widespread use of antibiotics in the last decades resulted in a rise of multidrug-resistant strains of both *E*. *coli* and *S*. *aureus*, thus, reducing the chances of a successful treatment of infections caused by these microorganisms. The increased bacterial resistance has driven the search for novel therapeutic approaches that can either replace the use or act as adjuvants of antibiotics [15]. In this context, plant-derived compounds have been widely investigated as antimicrobials and adjuvants to antibiotic therapy [16, 17].

The seeds of *Cuminum cyminum* L. (Apiaceae), popularly known as cumin, have been largely used in the global cuisine [18]. Its seeds are also used in the folk medicine to treat toothache, dyspepsia, diarrhea, epilepsy, amongst other disease conditions [18, 19]. Recent studies have demonstrated that the essential oil obtained from cumin seeds is antimicrobial against bacteria such as *S*. *aureus* and *E*. *coli* [20–27]. This activity has been attributed to the high content of cuminaldehyde in the cumin seed essential oil [18, 28, 29]. Recent studies have reported antimicrobial and anti-biofilm actions for this compound in *S*. *aureus* and *E*. *coli* strains [30–32]. In order to gain additional knowledge on cuminaldehyde properties, we investigated the *in silico* and *in vitro* activities of this compound against *S*. *aureus* and *E*. *coli*. We also assessed the ability of cuminaldehyde to potentiate ciprofloxacin effects on these bacteria.

## Materials and methods

### *In silico* analysis

#### Prediction of biological activities

To evaluate the biological activities of cuminaldehyde, the Prediction of Activity Spectra for Substances (PASS) Online programme was used. This computational tool calculates the probability of a given organic molecule to present a biological activity by comparing the molecule structure to a database (www.way2drug.com/passonline) composed of other organic molecules with defined biological activities [33, 34]. Thus, the PASS online programme gives the probability of a compound of being active (Pa) or inactive (Pi) on a biological target. For comparison, the biological activity of ciprofloxacin was also analyzed.

#### Prediction of oral bioavailability

The SwissADME programme (http://www.swissadme.ch/index.php#) [35] was used to predict the theoretical oral bioavailability of cuminaldehyde. For comparison, ciprofloxacin oral bioavailability was also assessed. Information on the following properties were obtained: total polar surface area (TPSA), partition coefficient (water/oil)—iLogP, molecular weight, number of hydrogen acceptors–nALH and number of hydrogen donors—nDLH of the compound. Then, an analysis based on the "Rule of Five" was performed as previously described [33, 36]. By definition, to present a good estimated oral bioavailability, a molecule needs to meet the requirements for at least three of the analyzed parameters: i) total polar surface area (TPSA) <140 A^**2**^, ii) LogP ≤ 5, iii) molecular weight <500 daltons, iv) number of acceptor hydrogen bonds (nALH) ≤ 10, and v) number of donor hydrogen bonds (nDLH) ≤ 5.

#### Estimation of pharmacokinetic characteristics and toxic effects

For analysis of the possible toxic effects and the theoretical pharmacokinetic parameters (absorption, distribution, metabolism and excretion) of cuminaldehyde, the Osiris (www.organic-chemistry.org/prog/peo/drugScore.html) [33] and SwissADME (http://www.swissadme.ch/index.php#) [35] programmes were used. These parameters and the toxicity were predicted by comparison of the cuminaldehyde chemical structure with a database containing commercially available drugs and commercially available compounds. Toxic effects were classified as mutagenic, tumorigenic, irritant, and effects on the reproductive system [33].

LD50 values in milligram per kilogram (mg/kg) were estimated by using the PROTOX programme (http://tox.charite.de/protox_II/index.php?site=compound_input) [37] and used to classify cuminaldehyde toxicity as follows: class i- fatal if swallowed at LD_**50**_ ≤ 5, class ii- fatal if swallowed when 5<LD_**50**_ ≤ 50, class iii- toxic if swallowed when 50 <LD_**50**_ ≤ 300, class iv- harmful if swallowed when 300 <LD_**50**_ ≤ 2000, class v- harmful if swallowed when 2000 <LD_**50**_ ≤ 5000, and class vi- non-toxic when LD_**50**_> 5000. Estimations of cuminaldehyde gastrointestinal absorption, permeability through the blood brain barrier and skin permeation (log Kp in centimeters (cm)/s) were assessed by the SwissADME programme (http://www.swissadme.ch/index.php#) [35]. Of note, the more negative the log Kp, the less skin permeant is the molecule.

Additionally, the probability of cuminaldehyde to becoming a commercial drug ("drug-score") was calculated by the Osiris programme (www.organic-chemistry.org/prog/peo/drugScore.html), by combining the values obtained for iLogP, drug-likeness, solubility (Log S; the closer the score is to 0, the higher the solubility) [35], molar mass and toxicity in to a single value. A drug-score value of 0.1 to 1.0 was taken as an index of suitability for commercialization [33].

### *In vitro* assays

#### Bacterial strains and culture

The standard strains of *S*. *aureus* ATCC 6538 and enteroaggregative *E*. *coli* 042 (EAEC 042), and the clinical isolates from urinary tract infections (*S*. *aureus*-A (Saa), *S*. *aureus*-V (Sav) and *E*. *coli*-R (Ecr)) were kindly provided by the bacterial collection sector of the Universidade CEUMA. The bacteria were cultured on Müeller-Hinton (MH; Merck Millipore) agar, at 37°C for 24 h as previously described [38]. The susceptibility profiles of the clinical isolates are depicted in the Table 1 and were determined in an automated VITEK® 2 system (BioMérieux Clinical Diagnostics, USA). Data interpretation was performed as recommended by the Clinical Laboratory Standards Institute—CLSI [39].

**Table 1:** Antibiotic susceptibility profiles of *Staphylococcus aureus* and *Escherichia coli* strains.

	Clinical isolate		
Antibiotic	*Saa*	*Sav*	*Ecr*
Amikacin	-	-	S
Amoxicillin/Clavulanate	-	-	R
Ampicillin	-	-	R
Cefepime	-	-	R
Ceftaroline	S	S	-
Ceftazidime	-	-	R
Ceftriaxone	-	-	R
Cefuroxime	-	-	R
Ciprofloxacin	S	S	R
Daptomycin	S	S	-
Ertapenem	-	-	S
Gentamicin	S	S	R
Imipenem	-	-	S
Levofloxacin	-	-	R
Linezolid	S	S	-
Meropenem	-	-	S
Minocycline	S	S	-
Nitrofurantoin	S	S	S
Norfloxacin	-	-	R
Oxacillin	S	S	-
Penicillin G	R	S	-
Piperacillin/Tazobactam	-	-	I
Rifampin	S	S	-
Teicoplanin	S	S	-
Tigecycline	-	-	-
Trimethoprim/Sulfamethoxazole	S	S	R
Vancomycin	S	S	-

R: resistant; S: susceptible; I: intermediate. Saa: *S*.*aureus*-A; Sav: *S*. *aureus*-V; Ecr: *E*. *coli*-R

#### Evaluation of minimum inhibitory concentrations (MICs)

Cuminaldehyde (98% purity; Sigma-Aldrich) antimicrobial effects were investigated by the microdilution method [40]. For this, the bacteria previously cultured on MH agar were suspended in saline (~ 1.5 × 10^**8**^ colony forming units (CFU)/milliliter (ml)). For determining the MICs, 10 μl of the bacterial suspension were incubated in MH broth (190 μl/well; Merck Millipore) containing different concentrations of cuminaldehyde (0.0234–24 mg/ml). Serial dilutions of ciprofloxacin (0.0009–200 μg/ml) were used as positive controls. Sterile dimethyl sulfoxide (DMSO, Sigma-Aldrich; 2% in saline) was used to increase cuminaldehyde solubility and as negative control. Samples were incubated for 24 h at 37°C, and the MICs (the lowest concentration at which no bacterial growth is observed) were evaluated. For this, the absorbances were read at 600 nm.

In parallel, the effects of the sub-inhibitory concentrations (MIC/2-MIC/8) of cuminaldehyde and ciprofloxacin on bacterial viability were assessed and calculated by addition of the PrestoBlue® reagent (1:10; Life Technologies), according to the manufacturer’s instructions. The absorbance was read at 570 nm and 600 nm and cell viability expressed as Δ absorbance in nm.

#### Effects on biofilm formation

Biofilm formation was quantified as previously described [38]. For this, 10 μl of each bacterial suspension (prepared as described above) were added per well into a 96-well cell culture plate containing sub-inhibitory concentrations of cuminaldehyde (MIC/2-MIC/8) or ciprofloxacin (MIC/2-MIC/8) in 190 μl of MH broth (Merck Millipore). Vehicle (2% DMSO in saline)-treated bacteria and broth without bacteria were used as negative controls. Samples were incubated at 37°C for 24 h, and then, the wells were washed three times with phosphate-buffered saline (PBS; Sigma-Aldrich). Samples were then, fixed with 200 μl of methanol (100%; Merck Millipore) for 15 min. Following, the methanol was removed and the plate wells allowed to air dry. Biofilm was stained with 5% crystal violet (Sigma-Aldrich) for 10 min at room temperature, and immediately solubilised with methanol (200 μl). The absorbance was read at 570 nm. Biofilm mass results are expressed as absorbance in nm. For analysis of biofilm viability, the PrestoBlue® reagent (1:10; Life Technologies) was used according to the manufacturer’s instructions. The absorbance was read at 570 nm and 600 nm and the results are expressed as Δ absorbance in nm.

#### Time-kill assay

An aliquot (200 μl) of each bacterial suspension was added to 2 ml of MH broth (Merck Millipore) containing ciprofloxacin (MIC/2) or cuminaldehyde (MIC/2-MIC/4) alone, or in combination. Vehicle (2% DMSO in saline)-treated bacteria were used as negative control. Cell growth was monitored by plating 10 μl of 10-fold-diluted suspensions from each sample at different time-points (0.15–8 h) in MH agar (Merck Millipore) plates. After 8 h of incubation at 37°C, the colonies were counted and then, the Log_**10**_ CFU/ml was calculated. The bactericidal combinatory effects were assessed by variation on Log_**10**_ CFU/ml (ΔLC). Synergy was defined as a decrease of ≥ 2 log_**10**_ CFU/ml and antagonism as an increase of #x2265; 2 log_**10**_ CFU/ml. If ΔLC was between 1 and 2 log_**10**_ CFU/ml, the effects were recorded as additive, and as indifferent if ΔLC = ± 1 log_**10**_ CFU/ml [40].

#### Synergy assay with ciprofloxacin on biofilm formation

The potential of cuminaldehyde to interact with ciprofloxacin was assessed on biofilm formation. Biofilm mass formation was quantified (as described above in the section **Effects on biofilm formation**). For this, 10 μl of each bacterial suspension were added per well into a 96-well cell culture plate containing cuminaldehyde (MIC/8) and ciprofloxacin (MIC/2) in 190 μl of MH broth (Merck Millipore). Vehicle (2% DMSO in saline)-treated bacteria and broth without bacteria were used as negative controls.

### Statistical analysis

All *in vitro* experiments were performed in triplicate and were obtained from three independent assays. Statistical comparison between groups was performed in the software GraphPad Prism version 5.0 by using one-way and repeated measures analysis of variance followed by the Bonferroni test. *P*<0.05 were considered significant.

## Results

### *In silico* analysis

#### Identified biological activities

Analysis of the probable biological activities of cuminaldehyde found that this compound has > 30% (Pa>0.3) probability to present 726 activities. Of those, 198 have moderate probability (Pa>0.5) of occurrence and 69, high probability of occurrence (Pa>0.7). Of the total identified activities, 15 were antimicrobial (Table 2).

**Table 2:** *In silico* identification of the antimicrobial activities of cuminaldehyde and ciprofloxacin.

Cuminaldehyde			Ciprofloxacin		
Antimicrobial activities	Pa value	Pi value	Antimicrobial activities	Pa value	Pi value
Inhibitor of *Porphyromonas gingivalis* Tpr proteinase	0.605	0.012	Ophthalmic antibacterial	0.940	0.000
Membrane permeability enhancer	0.516	0.012	Anti-infective	0.823	0.005
Antimycobacterial	0.507	0.018	DNA synthesis inhibitor	0.786	0.004
Antiparasitic	0.491	0.017	Topoisomerase II inhibitor	0.759	0.003
Anti-helmintic	0.487	0.006	Antimycobacterial	0.638	0.008
Antifungal	0.470	0.036	Antibacterial	0.589	0.009
Anti-nematode	0.445	0.028	Quinolone-like antibiotic	0.572	0.001
Anti-picornavirus	0.406	0.105	Anti-cytomegalovirus	0.448	0.004
DNA ligase (ATP) inhibitor	0.401	0.016	Anti-tuberculosis	0.452	0.019
Anti-*Helicobacter pylori*	0.381	0.011	DNA gyrase inhibitor	0.488	0.001
Membrane integrity antagonist	0.380	0.077	Antibiotic	0.358	0.010
Anti-infective	0.372	0.058	Anti-adenovirus	0.304	0.086
Anti-protozoal	0.352	0.060			
Anti-rhinovirus	0.349	0.160			
Antibacterial	0.336	0.047			

Pa: probability of a compound of being active; Pi: probability of a compound of being inactive.

For comparison, the biological activities of ciprofloxacin were also evaluated. Forty-seven activities with > 30% probability of occurrence were identified for ciprofloxacin. Of those, only 04 had high probability of occurrence (Pa>0.7). Analysis of all 47 biological activities indicated that 12 of them were antimicrobial (Table 2).

#### Estimated oral bioavailability and predicted toxicity

For predicting the oral bioavailibility of cuminaldehyde, its TPSA, iLogP, molecular weight, nALH and nDLH values were analyzed. Table 3 demonstrates that cuminaldehyde fits the criteria to present good estimated oral bioavailability (TPSA = 17.07; LogP of 2.03; molecular weight of 148.20; nALH of 1.0 and nDLH of 0.0). Ciprofloxacin presented a TPSA of 74.57, iLogP of 2.24, molecular weight of 331.34, nDLH of 2.0 and nALH of 5.0 (Table 3).

**Table 3:** *In silico* estimation of the oral bioavailability, toxic effects, absorption, solubility and drug-likeness score of cuminaldehyde in comparison with ciprofloxacin.

	Cuminaldehyde	Ciprofloxacin
**Estimated oral bioavailability**		
**iLogP**	2.03	2.24
**MW**	148.20	331.34
**TPSA**	17.07	74.57
**nDLH**	0	2
**Predicted toxic effects**		
**nADLH**	1	5
**Mutagenic effects**	None	High
**Tumorigenic effects**	None	None
**Irritant effects**	High	None
**Hepatotoxicity**	None	None
**Effects on reproduction**	None	None
**LD50 (mg/kg)**	1,320	2,000
**Toxicity class**	4	4
**Estimated absorption**		
**GI absorption**	High	High
**BBB permeability**	Yes	No
**Log K**_**p**_	-5.52 cm/s	-9.09 cm/s
**Predicted solubility and drug-likeness and score**		
**Log S**	-2.81	-3.32
**DL**	-11.1	2.07
**DS**	0.55	0.55

iLogP: partition coeficiente water: oil–lipophilicity index; MW: molecular weight; TPSA: total polar surface area; nALH: number of acceptor hydrogen bonds; nDLH number of donor hydrogen bonds; LD50: lethal dose 50%; GI: gastrointestinal absorption; BBB: blood brain barrier; Log Kp: skin permeation index; Log S: solubility; DL: drug-likness, DS: drug-score.

Table 3 depicts the predicted toxic effects of cuminaldehyde in comparison with those of ciprofloxacin. Cuminaldehyde was suggested to be an irritant, with no mutagenic, tumorigenic, hepatotoxic or harmful effects on reproduction. On the other hand, ciprofloxacin was found to be mutagenic, with no tumorigenic actions and no effects in the liver or the reproductive system. The estimated LD50 was 1,320 and 2,000 mg/kg for cuminaldehyde and ciprofloxacin; respectively (Table 3). Both drugs exhibited a toxicity score of 4.0, indicating they are classified as harmful if swallowed at their LD50 (Table 3).

Information on the estimations of gastrointestinal absorption, permeability through the blood brain barrier and skin permeation (log Kp in centimeters (cm)/s) are shown in Table 3. Both cuminaldehyde and ciprofloxacin were considered to be highly absorbed by the gastrointestinal tract; however, only cuminaldehyde was predicted to cross the blood brain barrier (Table 3). The estimated Log Kp values were of -5.52 and -9.09 cm/s for cuminaldehyde and ciprofloxacin; respectively (Table 3).

Table 3 also indicates the predicted solubility (LogS) of cuminaldehyde in comparison with ciprofloxacin. Both compounds were found to be soluble in water with Log S of -2.81 and -3.32 for cuminaldehyde and ciprofloxacin; respectively. Drug-likeness for cuminaldehyde was estimated at -11.01 and 2.07 for cuminaldehyde and ciprofloxacin; respectively. Both compounds presented similar drug-scores (0.55).

### Antimicrobial assays

#### Analysis of cuminaldehyde antimicrobial activity on *S*. *aureus* and *E*. *coli*

Table 4 shows the MIC values detected for cuminaldehyde and ciprofloxacin when assessed against the standard strains *S*. *aureus* ATCC 6538 and EAEC 042, and the clinical isolates Saa, Sav and Ecr. Cuminaldehyde presented antimicrobial activity against all tested bacteria, being more effective against EAEC 042 (1.5 mg/ml). All bacteria, except the clinical isolate Ecr, were susceptible to ciprofloxacin (Table 4).

**Table 4:** Minimum inhibitory concentration (MIC) values of cuminaldehyde in comparison with ciprofloxacin against *S*. *aureus* and *E*. *coli* strains.

	MIC	
Bacterial strain	Cuminaldehyde (mg/ml)	Ciprofloxacin (μg/ml)
***S*. *aureus* ATCC 6538**	12.0	0.0141
**Saa**	24.0	0.0141
**Sav**	24.0	0.225
**EAEC 042**	1.5	0.004
**Ecr**	12.0	100.0

Saa: *S*.*aureus*-A; Sav: *S*. *aureus*-V; Ecr: *E*. *coli*-R.

**Fig 1:**
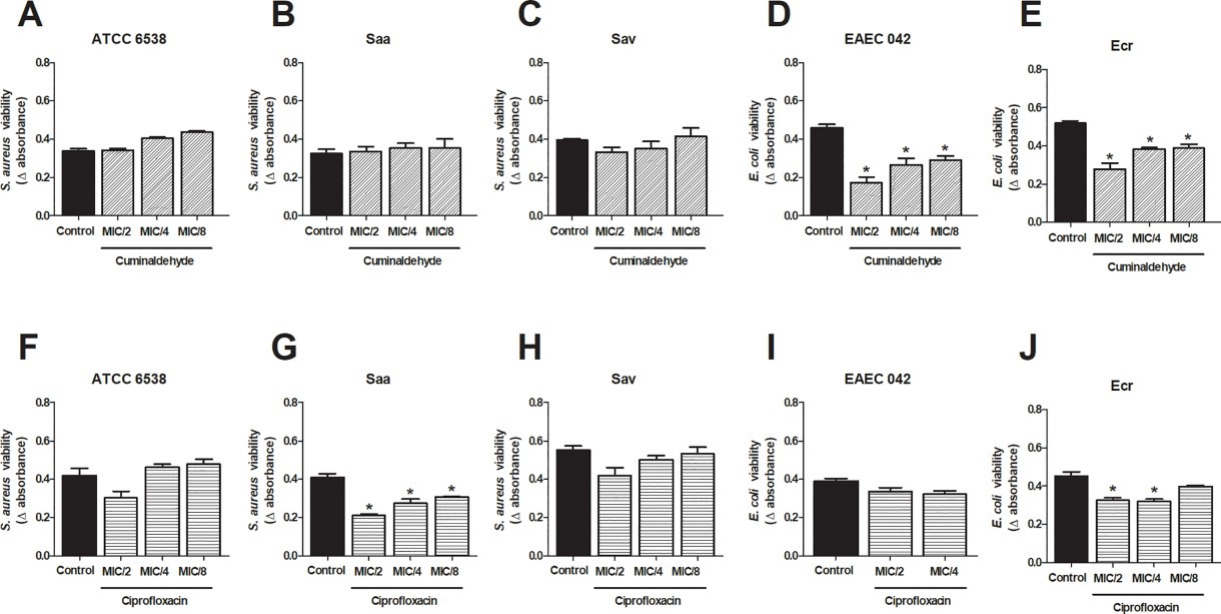
Effects of sub-inhibitory concentrations of cuminaldehyde in *S*. *aureus* and *E*. *coli* viability. Different cuminaldehyde concentrations (MIC/2-MIC/8) were incubated with *S*. *aureus* ATCC 6538 (a), EAEC 042 (e) and the clinical isolates Saa, Sav and Ecr (b,c and e), for 24h. For comparison, the effects of sub-inhibitory concentrations of ciprofloxacin were evaluated on *S*. *aureus* ATCC 6538 (f), EAEC 042 (i) and the clinical isolates Saa, Sav and Ecr (g,h and j). Vehicle (2% DMSO in saline)-treated bacteria were used as controls. *p<0.05; differs from the control group. Data were obtained from three independent experiments.

We additionaly investigated the effects of the sub-inhibitory concentrations of cuminaldehyde and ciprofloxacin. Whilst *E*. *coli* (EAEC 042 and Ecr; Fig 1D and 1E) viability was reduced (48–62%) by cuminaldehyde at MIC/2-MIC8, no effects were observed for these concentrations when assessed against *S*. *aureus* (Fig 1A–1C). Only the clinical isolates Saa and Ecr had their viability reduced by sub-inhibitory concentrations of ciprofloxacin (MIC/2-MIC/8; Fig 1F and 1J). Maximum reductions were of 49% and 30%, for Saa and Ecr; respectively.

#### Cuminaldehyde effects on biofilm formation by *S*. *aureus* and *E*. *coli*

**Fig 2:**
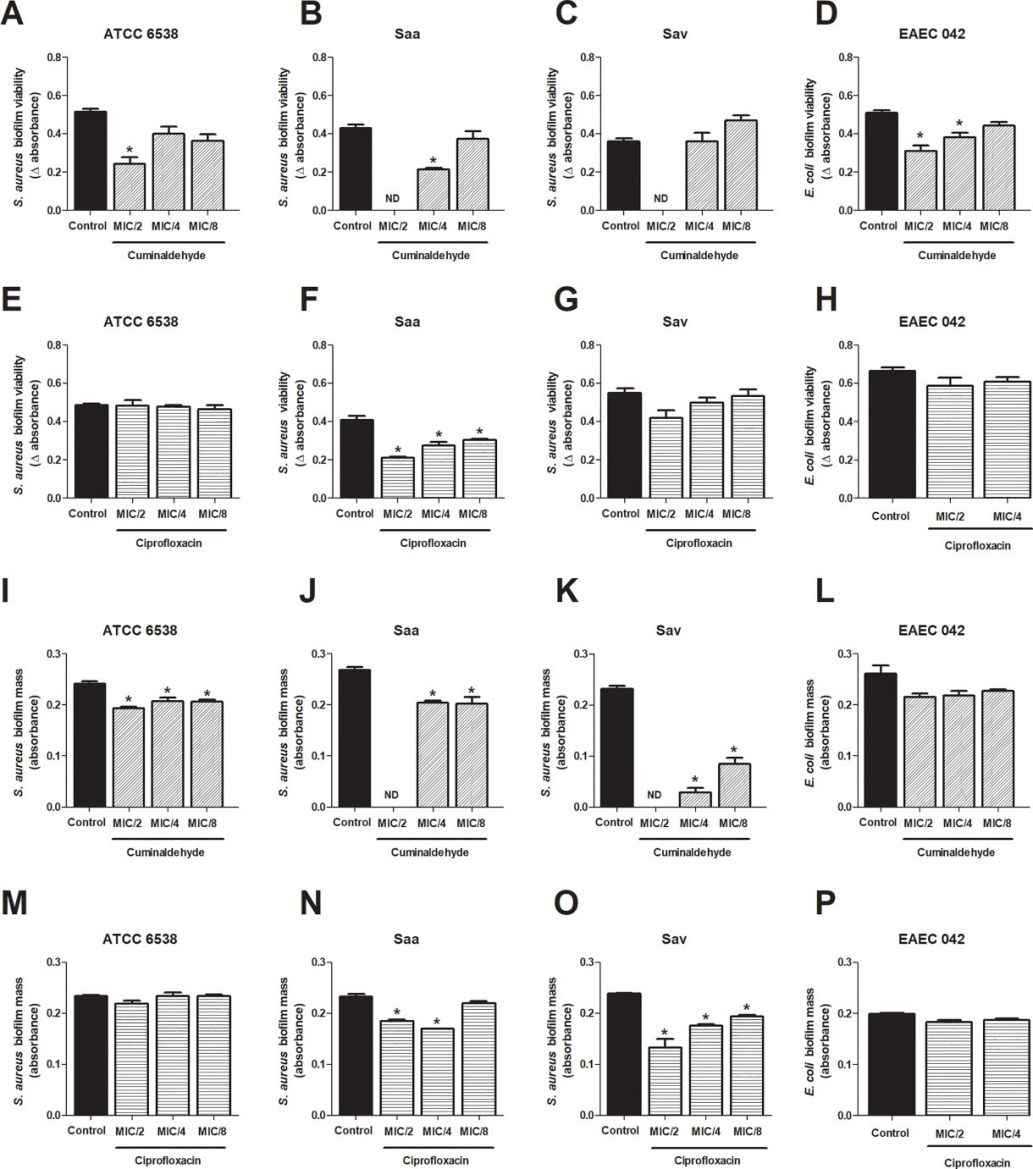
Cuminaldehyde *in vitro* effects on *S*. *aureus* and *E*. *coli* biofilm formation. Different concentrations of cuminaldehyde (MIC/2-MIC/8) were incubated with *S*. *aureus* and *E*. *coli*, for 24h. Biofilm viability was assessed Cuminaldehyde effects on the viability and mass of the biofilm formed by *S*. *aureus* ATCC 6538 (a and e), EAEC 042 (d and h) and the clinical isolates Saa (b and f) and Sav (c and g) were evaluated. For comparison, ciprofloxacin effects on the viability and mass of the biofilm formed by *S*. *aureus* ATCC 6538 (i and m), EAEC 042 (l and p) and the clinical isolates Saa (j and n) and Sav (k and o) were also assessed. Vehicle (2% DMSO in saline)-treated bacteria were used as controls. *p<0.05; differs from the control group. Data were obtained from three independent experiments.

The effects of the sub-inhibitory concentrations of cuminaldehyde on biofilm formation were investigated by analysis of biofilm mass and viability. All tested bacteria, except the clinical isolate Ecr, formed biofilm. Cuminaldehyde reduced the viability of bacterial biofilm when assessed at MIC/2 (Fig 2A–2D). Also, at MIC/4, the compound decreased the viability of both Saa and EAEC 042 (Fig 2B and 2D). On the other hand, only Saa biofilm viability was prevented by ciprofloxacin (MIC/2-MIC/8; Fig 2E–2H).

Also, cuminaldehyde significantly diminished biofilm mass formation by *S*. *aureus* (ATCC 6538, Saa and Sav; Fig 2I–2L). Ciprofloxacin inhibitory effects on biofilm mass were noted in the clinical isolates Saa and Sav (Fig 2N and 2O), but not in *S*. *aureus* ATCC 6538 and EAEC 042 strains (Fig 2M–2P).

#### Effects of the association of sub-inhibitory concentrations of cuminaldehyde and ciprofloxacin on bacterial survival

**Fig 3:**
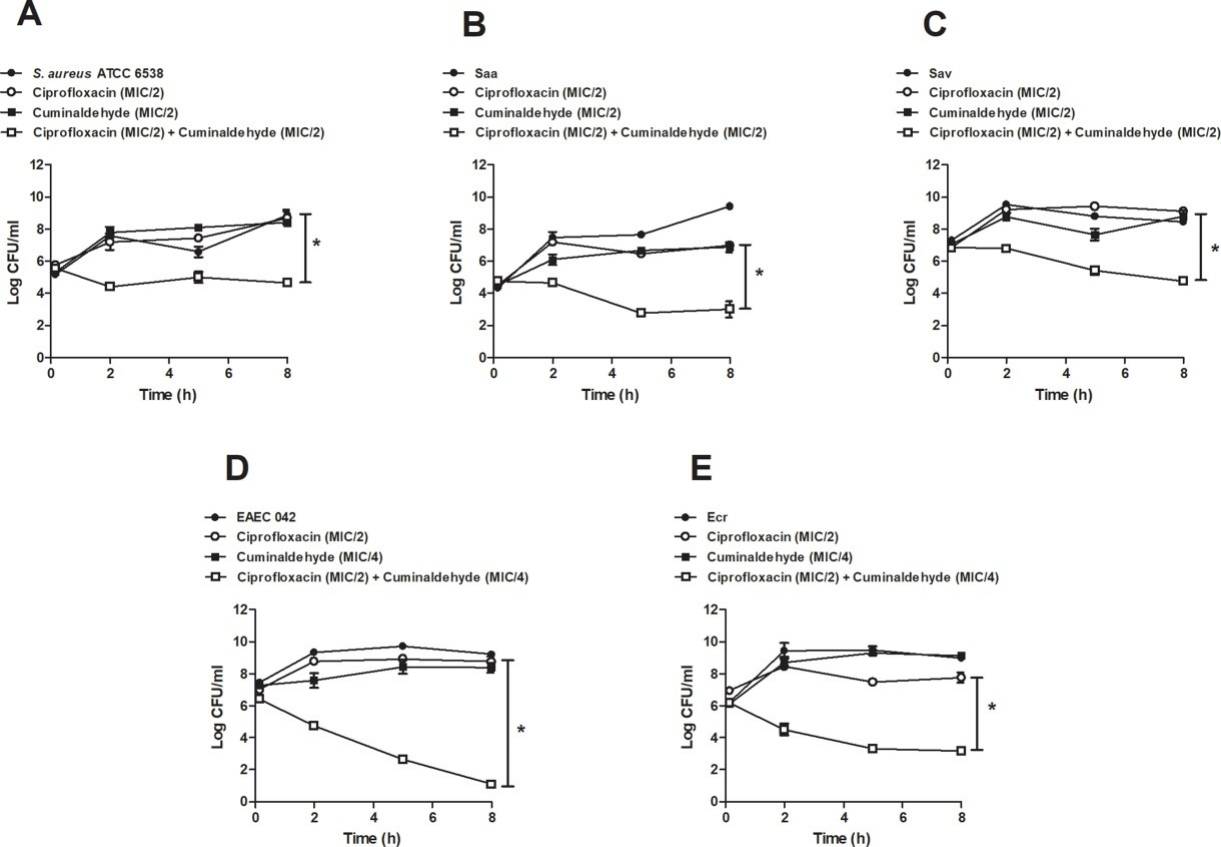
Effects of the co-incubation of cuminaldehyde and ciprofloxacin on *S*. *aureus* and *E*. *coli* survival. Cuminaldehyde (MIC/2 or MIC/4) was co-incubated with ciprofloxacin (MIC/2) over 8 h, and their effects were assessed against *S*. *aureus* ATCC 6538 (a), Saa (b), Sav (c), EAEC 042 (d) and (e) Ecr. Vehicle (2% DMSO in saline)-treated bacteria, as well those treated with either cuminaldehyde or ciprofloxacin were used as controls. *p<0.05; differs from the ciprofloxacin group. Data was obtained from 3 experiments.

Time-kill assays were performed to assess the effects of the association of cuminaldehyde with sub-inhibitory concentrations of ciprofloxacin on *S*. *aureus* and *E*. *coli* survival. The association of MIC/2 cuminaldehyde and MIC/2 ciprofloxacin was synergistic when assessed against *S*. *aureus*. Indeed, their association decreased bacterial population (> 4.0 log_*10*_ CFU/ml, > 3.0 log_*10*_ CFU/ml and > 4.0 log_*10*_ CFU/ml for *S*. *aureus* ATCC 6538, Saa and Sav; respectively; Fig 3A–3C). Similarly, the co-incubation of cuminaldehyde (MIC/4) with ciprofloxacin (MIC/2) significantly decreased *E*. *coli* survival (> 7.0 log_*10*_ CFU/ml.and (> 4.0 log_*10*_ CFU/ml, for EAEC 042 and Ecr; respectively; Fig 3D and 3E). No significant effects were observed for cuminaldehyde or ciprofloxacin by themselves, at the tested concentrations.

#### Effects of the association of sub-inhibitory concentrations of cuminaldehyde and ciprofloxacin on biofilm formation

**Fig 4:**
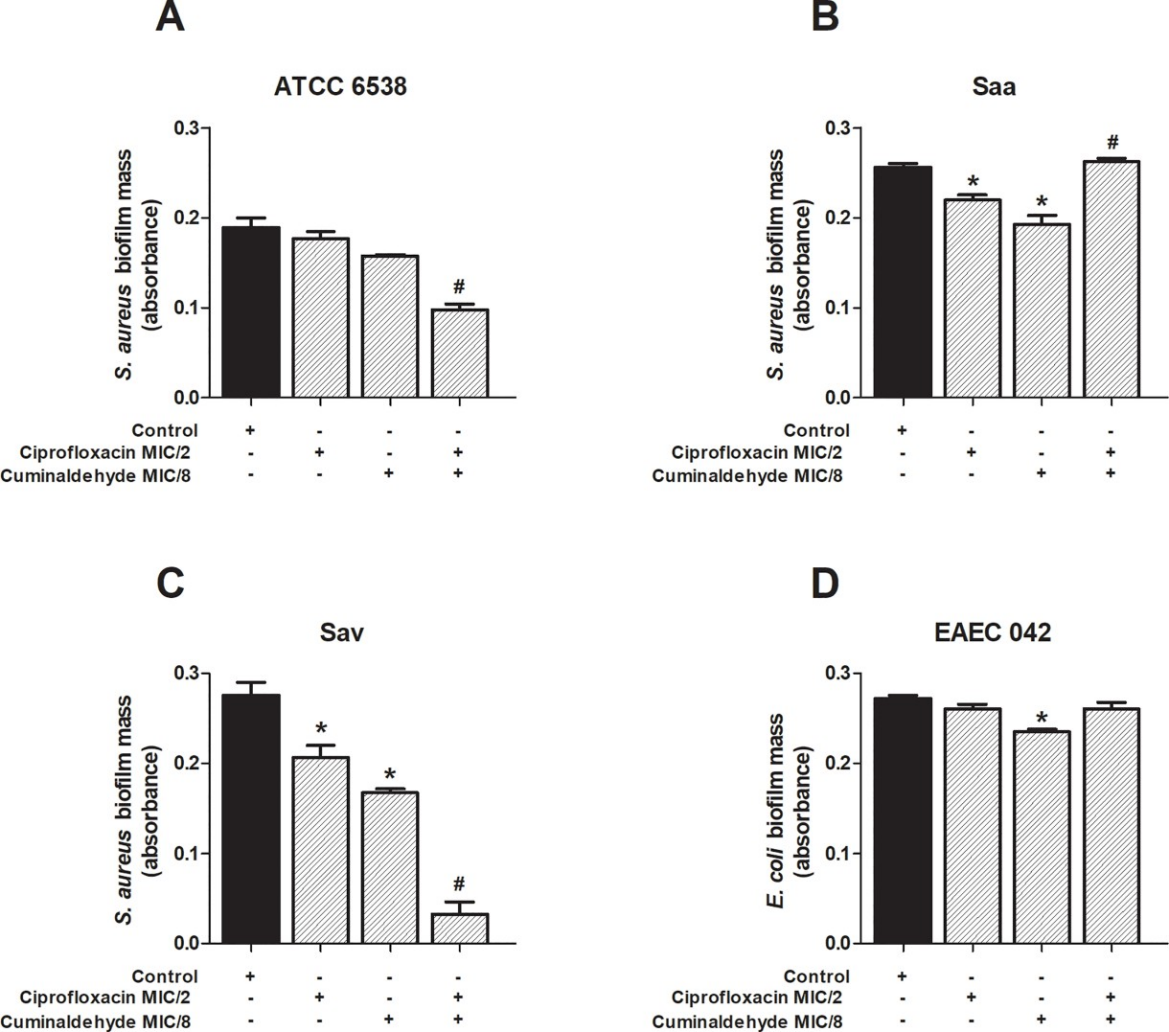
Effects of the co-incubation of cuminaldehyde and ciprofloxacin on *S*. *aureus* and *E*. *coli* biofilm mass formation. Cuminaldehyde (MIC/8) was co-incubated with ciprofloxacin (MIC/2), for 24h. The combination was assessed against *S*. *aureus* ATCC 6538 (a), EAEC 042 (d) and the clinical isolates Saa (b) and Sav (c). Vehicle (2% DMSO in saline), cumminaldehyde (MIC/8) and ciprofloxacin (MIC/2) effects on the biofilm mass formation were used as controls. *p<0.05; differs from the control group. #p<0.05; differs from the ciprofloxacin group. Data were obtained from three independent experiments.

The effects of the co-incubation of cuminaldehyde (MIC/8) and ciprofloxacin (MIC/2) sub-inhibitory concentrations were analysed (Fig 4A–4D). Data depicted in Fig 4 demonstrates that cuminaldehyde further reduces ciprofloxacin effects on the biofilm mass formation by *S*. *aureus* ATCC 6538 and Sav (panels a and c). However, it enhanced the amount of Saa-dependent biofilm formation (Fig 4B), without interfering with EAEC-induced biofilm (Fig 4D).

## Discussion

Studies with the essential oil of *C*. *cyminum* seeds indicated its antimicrobial potential against a series of bacteria including *S*. *aureus* and *E*. *coli* [20–27]. Cuminaldehyde has been pointed as the major compound associated with the pharmacological properties of *C*. *cyminum* seeds [18, 28].

In our study, the *in silico* evaluation of the biological activities of cuminaldehyde confirmed its antimicrobial potential. We aimed at gaining further knowledge of the anti-*S*. *aureus* and anti-*E*. *coli* actions of cuminaldehyde. Therefore, its properties and effects were compared to those of ciprofloxacin which has been commonly used for the treatment of urinary tract infections.

Evaluation of cuminaldehyde oral bioavailability estimated the compound is a good candidate for oral intake with a drug score of 0.55 (which indicates the probability of a compound of becoming a commercial drug), similar to the one observed for ciprofloxacin. However, doses >1,320 mg/kg of the compound would be lethal to mammalians, as cuminaldehyde was classified as harmful if swallowed at this LD50 (toxicity class equal to 4.0). *In silico* analysis also revealed that, as observed for ciprofloxacin, cuminaldehyde is likely to be absorbed by the gastrointestinal tract. Of note, this compound was suggested to be able to cross the blood brain barrier, meaning its dosing for systemic use should be carefully considered as it may result in unwanted effects in the nervous system.

Prediction of skin permeation (log Kp) indicated that cuminaldehyde is more likely to be absorbable by skin layers than ciprofloxacin. Of note, *S*. *aureus* is a common antibiotic-resistant pathogen colonizing skin wounds [41, 42]. Thus, topical cuminaldehyde may be useful as an adjuvant therapy to skin infections in association with topical antimicrobials. In this context, the predicted irritant effects of cuminaldehyde should be considered and a minimum effective dose should be adopted for its topical use.

Recent studies demonstrated cuminaldehyde has antimicrobial effects, presenting a MIC of 0.311 mg/ml and 0.650–4.98 mg/ml for *E*. *coli* and *S*. *aureus*; respectively [30,32,43]. In our study, cuminaldehyde was antimicrobial against all tested bacteria; however, it was found to be more effective against *E*. *coli*. Interestingly, the compound was antimicrobial against the ciprofloxacin-resistant clinical isolate Ecr. Analysis of sub-inhibitory concentrations of cuminaldehyde on bacterial viability reinforced its effectiveness against *E*. *coli*. Ciprofloxacin sub-inhibitory concentrations also reduced, although in a smaller degree, Ecr viability (30% viability reduction in ciprofloxacin *versus* 48% in cuminaldehyde-treated bacteria),

Of importance, compounds with anti-biofilm actions have been investigated as alternative therapies to treat bacterial infections [44–46]. It was previously reported that the essential oil of *C*. *cyminum* seeds reduces *Streptococcus mutans* and *S*. *pyogenes* biofilm formation [47]. Similar effects were observed for sub-inhibitory concentrations of the essential oil when assessed against *Klebsiella pneumoniae* [48]. Recently, cuminaldehyde was shown to impair biofilm formation by *E*. *coli* [32]. Herein, cuminaldehyde sub-inhibitory concentrations reduced the viability of all formed biofilms, but only significantly decreased *S*. *aureus* biofilm mass formation. By comparison, the sub-inhibitory concentrations of ciprofloxacin were only able to diminish Saa biofilm viability, in addition to Saa and Sav biofilm mass formation. These results indicate that cuminaldehyde presents a broader anti-biofilm activity than ciprofloxacin at the tested concentrations.

Many compounds have been investigated as adjuvants to antibiotic therapy to overcome bacterial resistance [16, 17]. The ability of *C*. *cyminum* seed essential oil to potentiate ciprofloxacin antimicrobial effects was shown against *K*. *pneumoniae* [48]. Herein, the *in silico* prediction of the biological activities of cuminaldehyde indicated this compound may act as a membrane permeability enhancer. Therefore, we hypothesized on whether this compound enhances the antimicrobial effects of ciprofloxacin. Our results demonstrate that cuminaldehyde (MIC/2-MIC/4) is synergistic to ciprofloxacin (MIC/2), as their association effectively reduces *S*. *aureus* and *E*. *coli* survival. A similar effect was seen in *S*. *aureus* (ATCC 6538 and Sav) biofilm formation. On the other hand, cuminaldehyde potentiated biofilm mass formation in ciprofloxacin-treated Saa, suggesting the combinatory effects of these drugs on biofilm mass formation may be strain-dependent.

Overall, the evidences gathered herein, suggest that cuminaldehyde may be useful as an adjuvant to ciprofloxacin therapy. As reduced doses of this commercial antibiotic would be used in conjunction with cuminaldehyde to treating *S*. *aureus* and *E*. *coli*-induced infections, lower pressure on bacterial resistance would probably result from this combined therapy; a hypothesis which remains to be investigated.

## Supporting information

S1 Rawdata (XLS)
